# Impact of Time to Surgery on Outcome in Wilms Tumor Treated with Preoperative Chemotherapy

**DOI:** 10.3390/cancers15051494

**Published:** 2023-02-27

**Authors:** Clemens-Magnus Meier, Rhoikos Furtwängler, Marvin Mergen, Nils Welter, Patrick Melchior, Jens-Peter Schenk, Christian Vokuhl, Leo Kager, Sabine Kroiss-Benninger, Stefan Wagenpfeil, Norbert Graf

**Affiliations:** 1 Department of General Surgery, Visceral, Vascular and Pediatric Surgery, Saarland University Medical Center, 66424 Homburg, Germany; 2 Department of Pediatric Oncology and Hematology, Saarland University Medical Center, 66424 Homburg, Germany; 3 Department of Radiation Oncology, Saarland University Medical Center, 66424 Homburg, Germany; 4 Pediatric Radiology Section, Department for Diagnostic and Interventional Radiology, University Hospital Heidelberg, 69120 Heidelberg, Germany; 5 Institute of Pathology, University Hospital Bonn, 53127 Bonn, Germany; 6 St. Anna Children’s Hospital, Department of Pediatrics, Medical University Vienna, 1090 Vienna, Austria; 7 St. Anna Children’s Cancer Research Institute, 1090 Vienna, Austria; 8 Department of Oncology, University Children’s Hospital Zürich, 8032 Zurich, Switzerland; 9 Institute for Medical Biometry, Epidemiology and Medical Informatics, Saarland University, Campus Homburg, 66424 Homburg, Germany

**Keywords:** Wilms tumor, preoperative chemotherapy, surgery, time to surgery, relapse-free survival, overall survival

## Abstract

**Simple Summary:**

In SIOP, trials and studies for Wilms tumor (WT) start with chemotherapy before the surgical removal of the tumor. The aim of this study is to find out whether the time between the start of preoperative chemotherapy and surgery has an influence on the outcome of WT patients. Therefore, we analyzed a completely German, Austrian, and Swiss cohort of 2561 unilateral WT patients pretreated between 1989 and 2022. This retrospective analysis shows no such influence on the occurrence of relapses and survival in unilateral WTs. However, in bilateral tumors with prolonged pretreatment, to achieve as many nephron-sparing surgeries as possible, preoperative treatment should not exceed 120 days due to an increased risk of relapses.

**Abstract:**

(1) Background: Wilms tumor (WT) treated preoperatively is cured in over 90% of cases. However, how long preoperative chemotherapy can be given is unknown. (2) Methods: 2561/3030 patients with WT (age < 18 years) treated between 1989 and 2022 according to SIOP-9/GPOH, SIOP-93-01/GPOH, and SIOP-2001/GPOH are retrospectively analyzed to assess the risk of time to surgery (TTS) for relapse-free survival (RFS) and overall survival (OS). (3) Results: TTS was calculated for all surgeries, with the mean being 39 days (38.5 ± 12.5) for unilateral tumors (UWT) and 70 days (69.9 ± 32.7) for bilateral disease (BWT). Relapse occurred in 347 patients, of which 63 (2.5%) were local, 199 (7.8%) were metastatic, and 85 (3.3%) were combined. Moreover, 184 patients (7.2%) died, 152 (5.9%) due to tumor progression. In UWT, recurrences and mortality are independent of TTS. For BWT without metastases at diagnosis, the incidence of recurrence is less than 18% up to 120 days and increases to 29% after 120 days, and to 60% after 150 days. The risk of relapse (Hazard Ratio) adjusted for age, local stage, and histological risk group increases to 2.87 after 120 days (CI 1.19–7.95, *p* = 0.022) and to 4.62 after 150 days (CI 1.17–18.26, *p* = 0.029). In metastatic BWT, no influence of TTS is detected. (4) Conclusions: The length of preoperative chemotherapy has no negative impact on RFS or OS in UWT. In BWT without metastatic disease, surgery should be performed before day 120, as the risk of recurrence increases significantly thereafter.

## 1. Introduction

Wilms tumor (WT) is the most common renal tumor in childhood. As a result of large prospective and randomized studies mainly conducted by the International Society of pediatric Oncology (SIOP) and the Children’s Oncology Group in North America (COG, formerly the National Wilms Tumor Study Group (NWTSG)), WT can now be cured in more than 90% of cases, despite different treatment strategies [1,2,3,4,5,6,7]. In contrast to COG, recommending primary surgery, the German Society of Hematology and Oncology (GPOH), part of SIOP, starts with preoperative chemotherapy (PC). PC leads to a decrease in tumor size and staging (downstaging) with less tumor ruptures and the omission of postoperative irradiation. In addition, a histologic response to treatment is used for postoperative treatment stratification [8].

Large studies have shown that overall survival is similar regardless of whether primary surgery or preoperative chemotherapy has been performed [2,6,9]. The SIOP 9 study’s aim was to investigate if a prolonged period (8 weeks) of preoperative chemotherapy could achieve a better local stage distribution. The results showed no significant difference in staging. Thus, the recommended preoperative treatment duration remained 4 weeks of Dactinomycin and Vincristine [10]. As the outcome in SIOP 9 was the same between 4 and 8 weeks of preoperative chemotherapy, such a prolonged preoperative treatment seems to be safe in nephroblastoma regarding oncological outcome. In bilateral WT (BWT), it is most important to reduce tumor size to achieve nephron-sparing surgery, at least on one side, in as many patients as possible. Therefore, preoperative treatment of up to 12 weeks is recommended today [7,11]. However, the question of for how long preoperative chemotherapy is safe is currently unanswered. This question is based on the suspicion that a tumor, even if treated with chemotherapy, may continue to spread or metastasize. 

Trying to answer this question, we retrospectively analyzed cohorts of WT from Germany, Austria, and Switzerland between 1989 and 2022 to see if there is a threshold for poorer outcomes related to a longer duration of preoperative chemotherapy.

## 2. Materials and Methods

This retrospective analysis is based on data from three successive studies and trials performed by the German Society of Pediatric Oncology and Hematology (GPOH) (SIOP-9/GPO, SIOP-93-01/GPOH and SIOP-2001/GPOH) for the treatment of children and adolescents with kidney tumors. These studies include prospectively enrolled patients from Germany, Austria, and Switzerland between 1989 and 2022 (up to 1 August 2022). All studies were reviewed and approved by the Ethics Committee of the Saarland Medical Board of Physicians (/LS from 23 April 1993, No. 136/01 from 20 September 2002 and, 248/13 from 13 January 2014). 

In this study, children and adolescents with WT receiving preoperative chemotherapy for minimum 1 and maximum 252 days (36 weeks) were included. The histology of WT after preoperative chemotherapy is classified according to SIOP [12,13]. Relapse was defined as a local recurrence and/or new metastasis. Local recurrence was defined as recurrence in the original tumor bed or the retroperitoneum, as well as within the abdominal cavity, the locoregional lymph node chain or the pelvis.

We investigated the impact of time to surgery (TTS) in terms of relapse-free survival (RFS) and overall survival (OS) according to Kaplan–Meier. To test whether the risk of relapse or death changed with increasing time from diagnosis to surgery, RFS and OS were calculated for different durations of preoperative chemotherapy. For unilateral tumors, patients were grouped according to TTS values of 35, 42, 49, 56, 63 and 70 days, and for bilateral tumors they were grouped according to TTS values of 90, 120 and 150 days. Statistical significance was calculated using the log-rank test.

To analyze the influence of TTS on the risk of relapse or death (hazard ratio, HR) univariate and multivariate Cox regression models with time-dependent covariate “time from diagnosis to surgery” were applied separately for unilateral and bilateral tumors, for patients without and with metastases at diagnosis, and for each interval of time. In addition to the HR and *p*-value, the 95% confidence interval (CI) is also reported. A Martingale residual plot was calculated and plotted for each of these groups to examine whether a time point could be identified at which the risk for relapse or death changed, and this was visualized by a locally estimated scatterplot smoothing (LOESS) line.

To account for possible confounding, the calculation was adjusted by the cofactors of age, local pathological stage, and histology (low-, intermediate-, high-risk). The cofactor of tumor volume after chemotherapy was not applied as it was not available in all cases (1851/2561). For the analysis of overall survival by Kaplan–Meier and Cox regression analyses, patients with a cause of death other than tumor progression (e.g., toxicity, other) were excluded.

Data on clinical and pathological outcomes were pseudonymized prior to statistical analysis. Computational and statistical analysis was performed using SPSS 27 for Mac (IBM SPSS Statistics 27, Armonk, NY, USA). Qualitative and quantitative values are presented as relative and absolute frequencies, as well as mean ± standard deviation, with median with interquartile range (IQR) in parentheses, respectively. The χ^2−^ test and Fischer exact test were used to compare relative frequencies between two independent groups. Quantitative values were compared using Mann–Whitney U tests. To compare quantitative variables for two dependent samples, the Wilcoxon test was used. Two-sided significance was defined as *p* < 0.05 for all the statistical tests. We did not account for the issue of multiple statistical testing due to the explorative nature of the investigation.

## 3. Results

### 3.1. General Results

Between 1989 and 2022, 3030 patients with Wilms tumor (WT) had been prospectively enrolled in the studies, and 2600 (85.8%) received preoperative chemotherapy (PC). Time to surgery (TTS) in 2565 patients with available data ranged from 1 to 252 days (mean 40.9 ± 17.1, median 36 (32/45)). Furthermore, 2367 tumors (92.3%) were unilateral and 194 were bilateral (7.6%). Four tumors (0.2%) were extrarenal, which were excluded from further analysis, and 483 (18.8%) of these patients already had metastases at diagnosis. The average age at diagnosis was 48 months (mean 47.8 ± 33.0, median 41 (23/63)) for unilateral and 32 months (mean 31.7 ± 22.1, median 28 (12/47)) for bilateral tumors (*p* < 0.01).

Surgery was performed after a mean of 38.5 ± 12.5 days (median 35 (IQR 31/43)) for unilateral tumors, after a mean of 35.4 ± 9.3 days (median 34 (IQR 31/38)) with no metastases at diagnosis, and after a mean of 51.6 ± 15.4 days (median 49 (IQR 44/56)) for unilateral WT (UWT) and metastatic disease. In the case of bilateral disease, patients were operated on after a mean of 69.9 ± 32.7 days (median 65 (IQR 43/91)), for BWT without metastasis it was after a mean of 70.7 ± 33.9 days (median 66 (IQR 43/92)), and in the case of metastasis it was after a mean of 66.2 ± 26.7 days (median 65 (IQR 48/86)) (Figure S1).

Altogether relapse occurred in 347 patients (13.5%), of which 308 were in UWT (88.8% of all relapses, representing 13.0% of unilateral tumors) and 39 were in BWT (11.2% of all relapses, representing 20% of bilateral tumors). Relapses were local in 63 (2.5%), metastatic in 199 (7.8%), and combined in 85 (3.3%) patients. 

**Figure 1:**
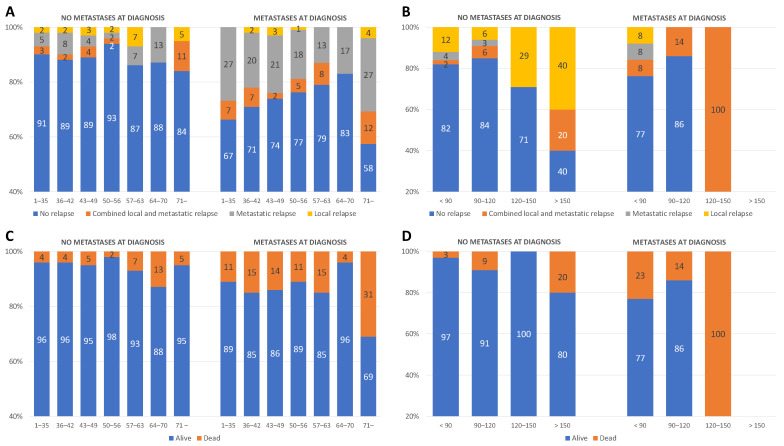
Relative incidence of relapse (**A**) and mortality (**C**) in unilateral WT and bilateral (**B**,**D**) WT without and with metastases at diagnosis and different preoperative treatment intervals.

As shown in Figure 1A, in unilateral WT without metastases at diagnosis, the number of recurrences is relatively constant with the increasing length of preoperative treatment. In contrast, in metastatic disease, the number decreases up to 70 days of preoperative therapy before increasing again. In this case, metastatic relapses predominate. In bilateral WT, relapses occurred with comparable frequency up to 120 days of preoperative therapy, respectively. After 120 days, the incidence increases substantially. This is true for patients without metastatic disease at diagnosis, and local recurrences dominate. In metastatic disease, the incidence decreased after 90 days. The only patient who underwent surgery after 120 days developed a relapse (Figure 1B). Relapses occurred after 15 months for unilateral disease (mean 15.1 ± 15.5, median 11 (7/17)) and after 21 months for bilateral disease (mean 21.1 ± 18.4, median 15 (9/28)) (For absolute numbers see Figure S2A,B).

During the study period, 184 patients (7.2%) died. In unilateral tumor without metastases, mortality is stable between 2 and 7% up to 64 days of preoperative chemotherapy. It doubles from 64–70 days to 13%, then decreases again to 5%. In primary metastatic disease, mortality is between 11 and 15% up to day 64, then drops to 4% by day 70, after which it rises again to one-third of cases (31%). In the presence of bilateral tumor without metastases at diagnosis, mortality is less than 10% up to 120 days, doubling to 20% after 150 days. For metastatic disease, mortality decreases up to 120 days, similar to the incidence of relapse. The only patient operated on after 120 days died (Figure 1C,D). The deaths were caused by tumor progression in 152 (82.6%) patients, toxicity in 15 (8.2%), unrelated to WT or its treatment in 12 (6.5%), and unknown in another 5 (2.7%) patients (For absolute numbers see Figure S2C,D).

For the whole cohort, a Kaplan–Meier (KM) analysis revealed an RFS of 87.0% for unilateral and of 79.9% for bilateral WT. RFS is significantly worse for patients with unilateral WT and metastatic disease (RFS = 74.2%) compared to those without metastasis (RFS = 90.0%) (*p* < 0.01). This is different for patients with bilateral disease. RFS is 80.6% for patients without metastasis compared to 76.5% for those with metastasis (*p* = 0.463) (Figure S3). The OS is 93% for unilateral WT and 89.2% for bilateral WT. It is significantly worse for patients with metastasis in unilateral (without: 96%, with: 86.3%; *p* < 0.001) and bilateral disease (without: 95.5%, with: 76.5%; *p* < 0.001) (Figure S4).

### 3.2. Influence of Time to Surgery on RFS

#### 3.2.1. Unilateral Tumors

**Figure 2:**
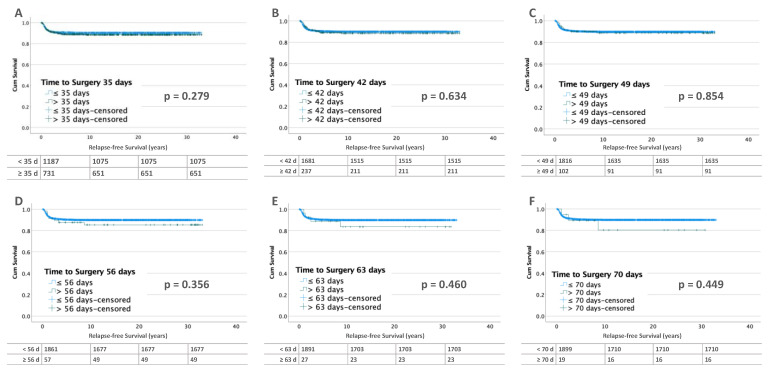
Kaplan–Meier lifetables of RFS of unilateral WT without metastases at diagnosis at thresholds of 35 (**A**), 42 (**B**), 49 (**C**), 56 (**D**), 63 (**E**) and 70 (**F**) days of preoperative chemotherapy. In the tables below the figures, the numbers at risk are reported.

Of the 2367 patients with unilateral WT, relapse occurred in 308 (13.0%). Lifetables calculated for the time points of 35, 42, 49, 56, and 63 days for patients without metastases at diagnosis (*n* = 1918) showed no differences in relapse-free survival (Figure 2). 

**Figure 3:**
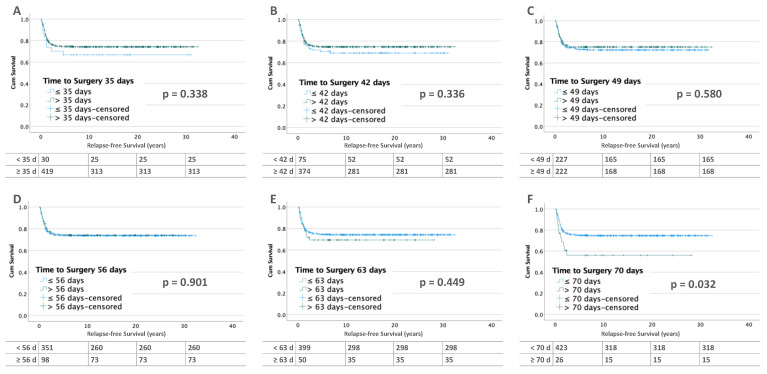
Kaplan–Meier lifetables of RFS of unilateral WT with metastases at diagnosis at thresholds of 35 (**A**), 42 (**B**), 49 (**C**), 56 (**D**), 63 (**E**) and 70 (**F**) days of preoperative chemotherapy. In the tables below the figures, the numbers at risk are reported.

This is different in patients with metastatic disease (*n* = 449) at diagnosis. After 70 days of preoperative chemotherapy, RFS was significantly worse (*p* = 0.032). RFS decreased from 75.2% (TTS ≤ 70 days) to 57.7% (Figure 3).

The Cox regression models confirmed the results of the lifetables for non-metastatic WT. In both univariate and multivariate analysis, no significant differences in RFS were found. In patients with metastatic disease at the 70-day time point, the risk of relapse was significantly increased (HR 1.96, CI 1.03–3.75, *p* = 0.041) in the univariate analysis, without confirmation in the multivariate analysis (Table 1).

In line with the Cox regression, Martingale residual plots (MRP) for patients with and without metastases at diagnosis also show no evidence that the risk of relapse changes during the period of preoperative chemotherapy (Figure S5a,b).

#### 3.2.2. Bilateral Tumors

**Figure 4:**
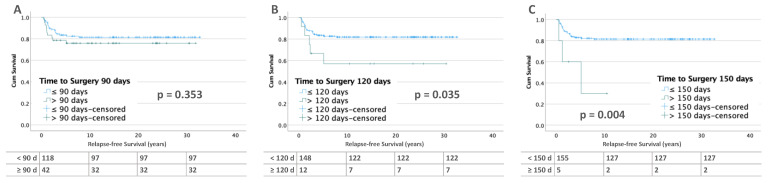
Kaplan–Meier lifetables of RFS of bilateral WT without metastases at diagnosis at thresholds of 90 (**A**), 120 (**B**), and 150 (**C**) days of preoperative chemotherapy. In the tables below the figures, the numbers at risk are reported.

**Figure 5:**
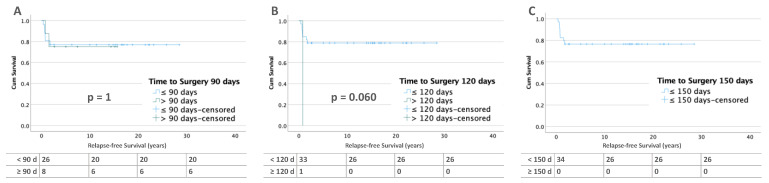
Kaplan–Meier lifetables of RFS of bilateral WT with metastases at diagnosis at thresholds of 90 (**A**), 120 (**B**), and 150 (**C**) days of preoperative chemotherapy. No patient underwent surgery after 150 days, so lifetable C shows only the <150-days group. In the tables below the figures, the numbers at risk are reported.

In bilateral WT (*n* = 194), 39 patients (21.1%) relapsed. Patients with bilateral tumor and no metastases at diagnosis show significantly worse RFS after 120 (58.3 %, *p* = 0.035) and 150 days (30%, *p* = 0.004) compared to those with less than 90 days (82.2 %, *p* = 0.353) of preoperative treatment (Figure 4). In contrast, there were no differences in RFS in patients with metastatic disease (Figure 5).

Using the Cox models, similar results are observed. Patients with bilateral tumor without metastasis at diagnosis are doing significantly worse for the time interval of 120 and 150 days. The hazard ratio (HR) in the univariate analysis for 120 days is 2.95 (CI 1.12–7.77, *p* = 0.029), and that for 150 days is 5.85 (CI 1.74–19.64, *p* = 0.004). The results at 120 and 150 days also persist in the multivariate analysis (HR 2.87, CI 1.19–7.95, *p* = 0.022 and HR 4.62, CI 1.17–18.26, *p* = 0.029). In the case of metastatic disease, no differences are found. However, this may also be due to the small number and the fact that only one surgery was performed after 120 days of preoperative therapy (Table 2).

The evaluation and visualization of the Martingale residuals result in a linear, almost horizontal, Loess line for the group of patients without metastatic disease, indicating no change in the risk of relapse. In this evaluation, only an interpretation up to 150 days is possible due to the small number of cases above 150 days. One case with a TTS of 225 days was excluded from the analysis (Figure S5c). The Martingale residual plot of bilateral patients with metastatic disease shows no change in risk for relapse (Figure S5d).

### 3.3. Influence of Time to Surgery on Overall Survival (OS)

#### 3.3.1. Unilateral Tumors

**Figure 6:**
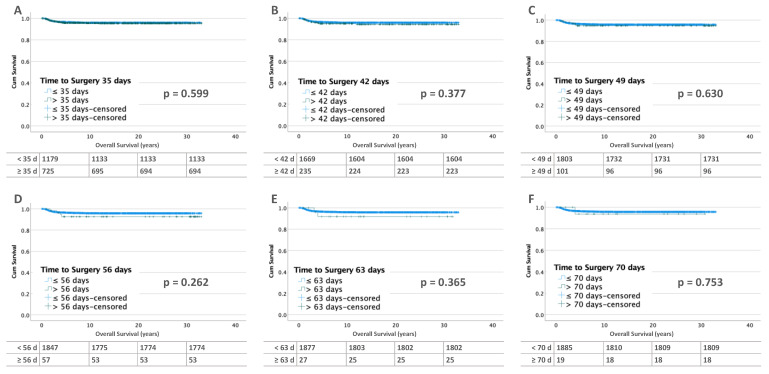
Kaplan–Meier lifetables of OS of unilateral WT without metastases at diagnosis at thresholds of 35 (**A**), 42 (**B**), 49 (**C**), 56 (**D**), 63 (**E**) and 70 (**F**) days of preoperative chemotherapy. In the tables below the figures, the numbers at risk are reported.

**Figure 7:**
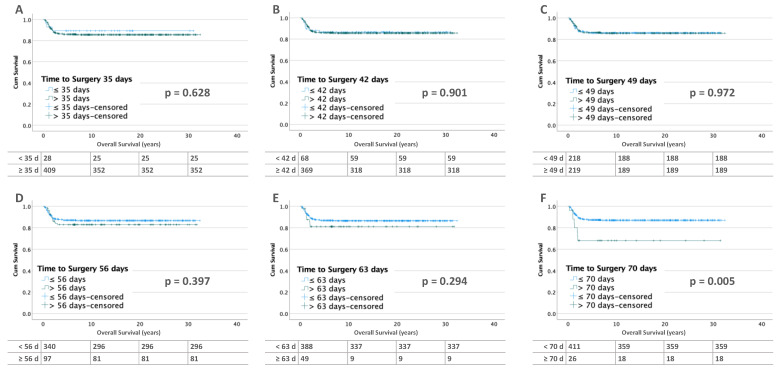
Kaplan–Meier lifetables of OS of unilateral WT with metastases at diagnosis at thresholds of 35 (**A**), 42 (**B**), 49 (**C**), 56 (**D**), 63 (**E**) and 70 (**F**) days of preoperative chemotherapy. In the tables below the figures, the numbers at risk are reported.

The overall survival for patients with unilateral tumor without metastases is not significantly different for the different time intervals (Figure 6). With metastatic disease, the intervals of 49, 56, 63, and 70 days show increasingly worse OS with a significant difference at 70 days (≤70 days: 87.3% vs. >70 days: 69.2%; *p* = 0.005) (Figure 7). 

These observations are confirmed by Cox models. For unilateral tumors without metastases, no differences in risk of recurrence are found. For metastatic disease, the univariate Cox model shows an increased HR of 2.77 (CI 1.31–5.84, *p* = 0.007) at 70 days (Table 3).

For overall survival, both unilateral tumors without and with metastases show a linear horizontal course of the Loess curve. This indicates a constant risk of death over time (Figure S6a,b).

#### 3.3.2. Bilateral Tumors

**Figure 8:**
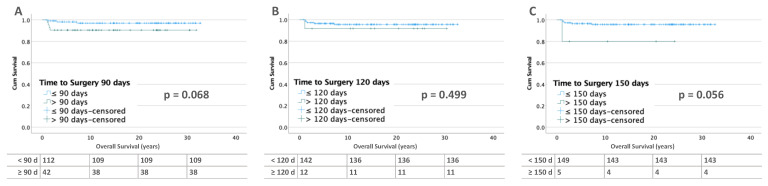
Kaplan–Meier lifetables of OS of bilateral WT without metastases at diagnosis at a thresholds of 90 (**A**), 120 (**B**), and 150 (**C**) days of preoperative chemotherapy. In the tables below the figures, the numbers at risk are reported.

**Figure 9:**
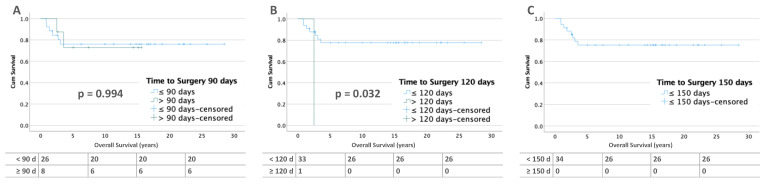
Kaplan–Meier lifetables of OS of bilateral WT with metastases at diagnosis at thresholds of 90 (**A**), 120 (**B**), and 150 (**C**) days of preoperative chemotherapy. No patient underwent surgery after 150 days, so lifetable C shows only the <150-days group. In the tables below the figures, the numbers at risk are reported.

For bilateral tumors without metastases at diagnosis, there is no difference in survival for the periods before 90 and 120 days. At 150 days, OS becomes non-significantly worse (≤150 days: 96% vs. >150 days: 80%; *p* = 0.056). This may be due to the small number of cases (*n* = 5) in this group (Figure 8). In bilateral WT with metastases, the OS for patients operated on before and after 90 days is between 70 and 80%. In addition, only one patient was operated on after 120 days and none after 150 days (Figure 9).

In the Cox analysis, there are no significant differences for OS in all groups. As in RFS, at the 120- and 150-day intervals the small number of cases prevent interpretation. (Table 4).

The Loess curve of Martingale residuals shows a linear and horizontal course for patients without metastases, indicating a constant unchanged risk of death (Figure S6c). This is different for patients with metastases. Here, the Loess curve is unsteady in the period from day 25 to 70 days of TTS, but without any significant rises or falls. Therefore, the risk is judged to be constant as well (Figure S6d).

## 4. Discussion

For decades, two opposing concepts have been used in the treatment of WT: primary surgery (PS) and preoperative chemotherapy (PC) [9]. Downstaging by PC has surgical advantages, making tumor resection safer and easier, and it may allow nephron-sparing surgery more often. In contrast, the early resection of the tumor avoids chemotherapy in the case of benign tumors [6,7]. Numerous studies have shown that the overall survival for PS and PC are identical [9,14,15]. Whether there is a threshold time to surgery where the outcome becomes worse with PC has not yet been investigated and is therefore unknown. In NWTSG, the time between surgery and start of postoperative radiotherapy was not found to be a significant prognostic parameter. A delay of 10 or more days showed the same recurrence rates at the flank and abdomen [16]. Nevertheless, after primary surgery, the early initiation of radiotherapy remains a critical component of multimodal treatment for patients with non-metastatic WT in NWTSG/COG. For non-metastatic patients, surgery to radiation intervals ≤ 14 days correlate with improved overall survival. However, no such association was noted for patients with metastases [17]. According to the analysis of 2565 patients, our retrospective analysis can be regarded as reliable. The only limitations of our study are the retrospective nature and the low number of patients with bilateral disease treated for more than 120 days preoperatively.

### 4.1. Unilateral Wilms Tumor 

Our results in unilateral WT show that the number of relapses decreases in absolute numbers with increasing preoperative treatment duration and does not increase in relative numbers per time interval. Regarding mortality, a similar result is obtained. This demonstrates that the prolongation of preoperative chemotherapy up to 70 days can be administered in unilateral WT with no impact on oncological outcome. Neither local nor metastatic relapses increase as a result of prolonged preoperative chemotherapy. This underlines the findings that local relapses are predominantly more dependent on local tumor extent and inadequate surgical procedures such as incomplete resection, intraoperative rupture, and no or inadequate lymph node dissection, than on the TTS itself [9,18]. Similar results are reported by Shamberger, who also blames surgical factors, such as tumor rupture, no lymph node dissection, or incomplete resection, for the occurrence of potentially avoidable local relapse [19].

In our cohort, half of unilateral WTs with primary metastatic disease were operated on before 49 days of TTS, which is in line with the 42 days of standard pretreatment duration. In this largest group, more than 50% of all relapses occurred. Treatment up to 70 days showed no increase in relapses. After 70 days of TTS, a significantly worse overall and relapse-free survival are shown in univariate but are not confirmed in multivariate analysis. This was partly due to locally very extended tumors where treatment was prolonged further, with the goal to achieve a resectable tumor status.

The length of preoperative chemotherapy has no impact on OS in unilateral tumors without metastases at diagnosis, according to the results of our study. In metastatic disease at diagnosis, only patients with a TTS of more than 70 days show a significantly worse OS and RFS in univariate, but not in multivariate Cox regression. One reason why this outcome was not produced from the multivariate analysis may be the personalized, individualized risk-adapted treatment that outweighs the pure effect of the time of preoperative treatment. Another possible reason could be that seven of eight patients with relapse had additional risk factors, such as an age older than 48 months and diffuse anaplasia (in two patients). In this analysis, no tumor volume and molecular data are included, so their influence is unknown during the prolongation of TTS.

Our trials achieved an RFS of 87.0% for all WT patients, whereas the presence of metastases at diagnosis is associated with a worse outcome, of 74.2% (RFS in metastatic patients vs. 90.0% in non-metastatic patients). Comparable results are also reported after primary surgery by other trial groups [9,14,15]. The advantages of PC are not outweighed by the poorer outcome in comparison to PS [18,19,20].

### 4.2. Bilateral Wilms Tumor 

Bilateral WTs are diagnosed at a younger age than unilateral and account for only 5–10% of cases. In contrast to unilateral WTs, bilateral WTs are more commonly associated with cancer predisposition syndromes (e.g., WAGR syndrome, Denys-Dash syndrome, and the Beckwith–Wiedemann spectrum) and are frequently multifocal, and recurrence occurs early [8,21,22,23,24]. The treatment of bilateral WTs is more complex, and a prolonged response-adapted PC is the gold standard today to spare as much healthy renal tissue as possible with nephron-sparing surgeries, to avoid end-stage renal failure. Therefore, as long as chemotherapy for a maximum of up to 12 weeks induces a response in tumor volume, PC is justified. However, in the case of nephroblastomatosis, prolonged preoperative treatment results in a worse outcome, comparable with that of patients with metastatic disease [25]. In our cohort, bilateral tumors were pretreated for twice as long as unilateral tumors. Internationally, 12 weeks of preoperative chemotherapy is now regarded as the standard time interval before surgery. This is also consistent with Kieran’s suggestion to not treat for longer than 12 weeks, as longer therapy is associated with increased therapy-associated morbidity but not improved survival [8]. In our cohort, the relative frequency of relapses is stable between 18% (<90 days) and 15% (90–120 days) and increases at 120 days to 29% and to 60% at 150 days. The risk of relapse (HR), adjusted for age, local stage, and histological risk group, shows an increase of 2.87 (CI 1.19–7.95, *p* = 0.022) after 120 days and even of 4.62 (CI 1.17–18.26, *p* = 0.029) after 150 days. In primary metastatic disease, eight patients (*n* = 8/34, 23.5%) underwent surgery after 90 days, and one of these was after 120 days, thus not allowing further statistical analysis. 

Nephron-sparing surgery is the preferred approach in SIOP, as well as in COG [9], in patients with bilateral disease aiming at optimal oncologic control and maximum preserved renal function. PC facilitates nephron-sparing procedures, at least on one kidney in most cases [4,9,26,27,28,29]. Nevertheless, the long-term rate of end-stage renal disease for bilateral WT still approaches 12–15% [27,30]. The incidence of relapse after NSS in bilateral tumors is reported to be between 12 and 29% [4,26,29,30,31,32]. Thus, the frequency of relapse in our cohort of 20% is within this range. In contrast to unilateral tumors, relapses in bilateral tumors are more often local. In patients without primary metastases, two-thirds of the relapses occur locally in the patients being operated on before 90 days of TTS. Some of the reasons may be multifocal disease and nephrogenic rests in the remaining kidney, both of which are more common in bilateral WT [31]. It is often difficult to distinguish between the growth of a residual tumor or a de novo tumor, but both are independent of TTS.

The OS for bilateral WT without metastatic disease at diagnosis is 90% up to 120 days of preoperative chemotherapy. Although the incidence of relapses increases to 29% at 120 days and then to 60% at 150 days, mortality increases insignificantly to 20% at 150 days. TTS shows no negative influence on OS (80%) despite the increase of relapses. Late surgeries are always performed where the resection of the tumor is difficult and the prolongation of chemotherapy is thought to allow easier and better nephron-sparing surgery, which is rarely the case. This and the remaining nephrogenic rests after surgery can explain the high rate of relapses. In pre-existing metastatic disease, OS remains stable at just below 80% up to 120 days of preoperative chemotherapy. Hamilton reported results from NWTS-4 of an EFS of 63% and an OS of 84% for bilateral WT, which is in line with our results [30]. In the first prospective study in children with BWT of COG (AREN0534), four-year EFS and OS were 82.1% (95% CI: 73.5–90.8%) and 94.9% (95% CI: 90.1–99.7% [33]. The reported five-year EFS and OS rates from the French SIOP-93-01 trial are 83.4% and 89.5%, respectively [32]. Seven out of forty-nine patients developed a relapse. All relapses occurred after preoperative chemotherapy of more than 120 days, which lasted for a median of 80 days. The duration of the whole chemotherapy was between 134 and 599 days. Four of these patients died.

According to our data, TTS intervals in patients undergoing preoperative chemotherapy have no negative influence on the outcome in children with nephroblastoma, underlining that there is no risk of tumor spread during a prolonged preoperative treatment. There are only a few studies on childhood tumors analyzing the effect of the prolongation of preoperative treatment. Rojas et al. studied the optimal timing of resection in high-risk neuroblastoma. They compared OS after two, three, four, and five cycles of chemotherapy [34]. There was an improvement in outcome from two to four cycles (33%, 45%, 83%). After five cycles, this was significantly worse (36%, *p* = 0.07), but multivariate analysis showed significantly lower mortality after four versus two cycles (*p* = 0.04). The worse outcome at five cycles was due to toxicity. Event-free survival did not differ with respect to the number of cycles. The authors concluded that the best time to perform tumor resection is after four cycles.

More studies are available on adult tumors, such as breast cancer, posing that there is a significant worsening of survival when surgery is delayed. Bleicher et al. studied the treatment courses of 95,000 patients (SEER-Medicare data) older than 66 years with breast cancer. Overall survival was reduced with the increasing delay of surgery (TTS ≤ 30, 31–60, 61–90, 91–120, 121–180 days; *p* < 0.001). Breast cancer-specific mortality also increased with each 60-day interval (*p* = 0.03) [35]. In the same paper [35], another National Cancer Data Base (NCBD) study of 115,000 patients older than 18 years was described by the authors, explaining that overall mortality increased with each time interval (*p* < 0.001) [35]. The same is true for other tumor types, such as colon carcinoma. Kaltenmeier et al. analyzed the data of 514,000 patients with colon cancer from the National Cancer Data Base (NCDB). There was a significant increase in mortality, with TTS < 7 (HR 1.56, CI 1.45–168, *p* < 0.001), 31 to 60 (HR 1.13, CI 1.02–1.25, *p* = 0.0018), 61 to 90 (HR 1.49, CI 1.19–1.85, *p* < 0.001), 91 to 120 (HR 2.28, CI1.61–3.23, *p* < 0.001), and 121 to 180 days (HR 2.46, CI 1.04–1.05, *p* < 0.001) compared with surgery 7 to 30 days after diagnosis [36].

## 5. Conclusions

Preoperative chemotherapy and thus delayed surgery has no negative influence on RFS or OS in unilateral, localized WT. However, in bilateral WT without metastatic disease, surgery should be performed before day 120, as the risk of relapse increases significantly thereafter. This analysis confirms that the current preoperative treatment duration in SIOP protocols for unilateral WT can be applied without an oncological risk and that for bilateral WT, preoperative treatment should not exceed the current recommendation of 12 weeks. 

## Figures and Tables

**Table 1:** Results of univariate und multivariate regression analysis of the impact of TTS on RFS at different time points for patients with unilateral WT with and without metastases at diagnosis.

TTS (Days)		Relapse					Univariate Cox Regression			Multiple Cox Regression		
		No		Yes		χ^2^	HR	CI	*p*	HR	CI	*p*
**Without Metastases at Diagnosis (*n* = 1918)**												
35	≤35	1075	90.6%	112	9.4%	0.309	1.16	0.87–1.55	0.319	1.02	0.76–1.38	0.896
	>35	651	89.1%	80	10.9%							
42	≤42	1515	90.1%	166	9.9%	0.643	1.08	0.71–1.64	0.706	0.95	0.62–1.45	0.801
	>42	221	89.0%	26	11.0%							
49	≤49	1635	90.0%	181	10.0%	0.865	1.02	0.55–1.89	0.947	0.92	0.49–1.70	0.784
	>49	91	89.2%	11	10.8%							
56	≤56	1677	90.1%	184	9.9%	0.366	1.33	0.65–2.73	0.441	1.09	0.53–2.26	0.814
	>56	49	86.0%	8	14.0%							
63	≤63	1703	90.1%	188	9.9%	0.512 *	1.34	0.48–3.70	0.574	1.03	0.37–2.90	0.950
	>63	23	85.2%	4	14.8%							
70	≤70	1710	90.0%	189	10.0%	0.428 *	1.40	0.43–4.55	0.573	1.07	0.32–3.52	0.918
	>70	16	84.2%	3	15.8%							
**With Metastases at Diagnosis (*n* = 449)**												
35	≤35	20	66.7%	10	33.3%	0.387	0.80	0.40–1.58	0.518	1.07	0.53–2.15	0.853
	>35	313	74.7%	106	25.3%							
42	≤42	52	69.3%	23	30.7%	0.313	0.82	0.51–1.31	0.414	1.02	0.62–1.66	0.948
	>42	281	75.1%	93	24.9%							
49	≤49	165	72.7%	62	27.3%	0.518	0.90	0.62–1.30	0.566	0.84	0.56–1.26	0.397
	>49	168	75.7%	54	24.3%							
56	≤56	260	74.1%	91	25.9%	1	0.95	0.60–1.50	0.816	0.82	0.49–1.37	0.448
	>56	73	74.5%	25	25.5%							
63	≤63	298	74.7%	101	25.3%	0.494	1.20	0.69–2.12	0.515	0.90	0.46–1.77	0.751
	>63	35	70.0%	15	30.0%							
70	≤70	318	75.2%	105	24.8%	0.063	1.96	1.03–3.75	**0.041**	1.44	0.64–3.23	0.375
	>70	15	57.7%	11	42.3%							

* Because of a number of 5 or less than 5 in at least one category, the Fisher Exact Test was used. TTS: Time to surgery. Numbers in bold are significant.

**Table 2:** Results of univariate und multivariate regression analysis of the impact of TTS on RFS at different time points for patients with bilateral WT with and without metastases at diagnosis. After 150 days of pretreatment, only 5 patients are included.

TTS (Days)		Relapse					Univariate Cox Regression			Multiple Cox Regression		
		No		Yes		χ^2^	HR	CI	*p*	HR	CI	*p*
**Without Metastases at Diagnosis (*n* = 160)**												
90	<90	97	82.2%	21	17.8%	0.495	1.48	0.69–3.15	0.313	1.22	0.56–2.66	0.618
	≥90	32	76.2%	10	23.8%							
120	<120	122	82.4%	26	17.4%	0.057 *	**2.95**	**1.12–7.77**	**0.029**	**2.87**	**1.19–7.95**	**0.022**
	≥120	7	58.3%	5	41.7%							
150	<150	127	81.9%	28	18.1%	0.050 *	**5.85**	**1.74–19.64**	**0.004**	**4.62**	**1.17–18.26**	**0.029**
	≥150	2	40%	3	60%							
**With Metastases at Diagnosis (*n* = 34)**												
90	<90	20	76.9%	6	23.1%	1 *	1	0.20–4.96	1	1.04	0.16–6.90	0.966
	≥90	6	75.0%	2	25.0%							
120#	<120	26	78.8%	7	21.2%	-	-	-	-	-	-	-
	≥120	-	-	1	100%							
150#	<150	26	76.5%	8	23.5%	-	-	-	-	-	-	-
	≥150	-	-	-	-							

* Because of a number of 5 or less than 5 in at least one category, the Fisher Exact Test was used. # No regression analysis was performed here because one of the groups included only one or no patients. TTS: Time to surgery. Numbers in bold are significant.

**Table 3:** Results of univariate und multivariate regression analysis of the impact of TTS on OS at different time points for patients with unilateral WT with and without metastases at diagnosis.

TTS (Days)		Death					Univariate Cox Regression			Multiple Cox Regression		
		No		Yes		χ^2^	HR	CI	*p*	HR	CI	*p*
**Without Metastases at Diagnosis (*n* = 1904)**												
35	≤35	1133	96.1%	46	3.9%	0.720	1.13	0.72–1.78	0.605	1.02	0.64–1.63	0.944
	>35	694	95.7%	31	4.3%							
42	≤42	1604	96.1%	65	3.9%	0.480	1.35	0.73–2.50	0.341	0.98	0.51–1.89	0.960
	>42	223	94.9%	12	5.1%							
49	≤49	1731	96.0%	72	4.0%	0.600 *	1.25	0.50–3.09	0.633	1.04	0.42–2.57	0.938
	>49	96	95.0%	5	5.0%							
56	≤56	1774	96.0%	73	4.0%	0.288 *	1.76	0.64–4.82	0.270	1.23	0.45–3.39	0.688
	>56	53	93.0%	4	7.0%							
63	≤63	1802	96.0%	75	4.0%	0.624 *	1.89	0.46–7.70	0.374	1.35	0.33–5.51	0.681
	>63	25	92.6%	2	7.4%							
70	≤70	1809	96.0%	76	4.0%	0.545 *	1.37	0.19–9.85	0.755	1.06	0.15–7.69	0.951
	>70	18	94.7	1	5.3%							
**With Metastases at Diagnosis (*n* = 437)**												
35	≤35	25	89.3%	3	10.7%	0.783 *	1.33	0.42–4.23	0.635	2.07	0.64–6.71	0.224
	>35	352	86.1%	57	13.9%							
42	≤42	59	86.8%	9	13.2%	1	1.04	0.51–2.12	0.905	1.81	0.85–3.82	0.121
	>42	318	86.2%	51	13.8%							
49	≤49	188	86.2%	30	13.8%	1	1.01	0.61–1.67	0.974	1.05	0.60–1.84	0.878
	>49	189	86.3%	30	13.7%							
56	≤56	296	87.1%	44	12.9%	0.403	1.28	0.72–2.27	0.399	0.88	0.45–1.72	0.700
	>56	81	83.5%	16	16.5%							
63	≤63	337	86.9%	51	13.1%	0.376	1.46	0.72–2.96	0.297	0.89	0.35–2.29	0.813
	>63	40	81.6%	9	18.4%							
70	≤70	359	87.3%	52	12.7%	**0.017**	**2.77**	**1.31–5.84**	**0.007**	1.32	0.46–3.78	0.611
	>70	18	69.2%	8	30.8%							

* Because of a number of 5 or less than 5 in at least one category, the Fisher Exact Test was used. TTS: Time to surgery. Numbers in bold are significant.

**Table 4:** Results of univariate und multivariate regression analysis of the impact of TTS on OS at different time points for patients with bilateral WT with and without metastases at diagnosis.

TTS (Days)		Death					Univariate Cox Regression			Multiple Cox Regression		
		No		Yes		χ^2^	HR	CI	*p*	HR	CI	*p*
**Without Metastases at Diagnosis (*n* = 154)**												
90	<90	109	97.3%	3	2.7%	0.089 *	3.64	0.81–16.23	0.091	1.31	0.25–6.91	0.754
	≥90	38	90.5%	4	9.5%							
120	<120	136	95.8%	6	4.2%	0.440 *	2.03	0.24–16.87	0.512	9.65	0.58–166.11	0.115
	≥120	11	91.7%	1	8.3%							
150	<150	143	96.0%	6	4.0	0.210 *	6.07	0.73–50.51	0.095	12.23	0.69–215.48	0.087
	≥150	4	80%	1	20%							
**With Metastases at Diagnosis (*n* = 34)**												
90	<90	20	76.9%	6	23.1%	1 *	1	0.20–4.96	1	1.11	0.21–5.72	0.905
	≥90	6	75%	2	25%							
120#	<120	26	78.8%	7	21.2%	0.235 *	-	-	-	-	-	-
	≥120	-	-	1	100%							
150#	<150	26	76.5	8	23.5%	-	-	-	-	-	-	-
	≥150	-	-	-	-							

* Because of a number of 5 or less than 5 in at least one category, the Fisher Exact Test was used. # No regression analysis was performed here because one of the groups included only one or no patients. TTS: Time to surgery.

## Data Availability

The data presented in this study are available on request from the corresponding author. The data are not publicly available due to ongoing analysis.

## Supplementary Materials

The following supporting information can be downloaded at: www.mdpi.com/article/10.3390/cancers15051494/s1, Figure S1:TTS of unilateral and bilateral WT depending on metastases at diagnosis; Figure S2: Frequency of relapse (coloured, A/B) and mortality (grey, C/D) in unilateral and bilateral WT without and with metastases at diagnosis and different preoperative time intervals; Figure S3: Kaplan–Meier lifetables of RFS of unilateral (A/C) and bilateral (B/D) WT and depending of metastases at diagnosis; Figure S4: Kaplan–Meier lifetables of OS of unilateral (A/C) and bilateral (B/D) WT and depending of metastases at diagnosis; Figure S5: Martingale residual plots of RFS for unilateral (A/B) and bilateral WT (C/D) with and without metastases at diagnosis; Figure S6: Martingale residual plots of OS for unilateral (A/B) and bilateral WT (C/D) with and without metastases at diagnosis.
