# Supplementary material for: Impact of Time to Surgery on Outcome in Wilms Tumor Treated with Preoperative Chemotherapy
Source: Cancers (Basel). 2023 Feb 27;15(5):1494. doi: 10.3390/cancers15051494 (PMC10001069; doi:10.3390/cancers15051494)

Wilms Tumors - Time to Surgery in days

Without metastases at diagnosis

With metastases at diagnosis

Unilateral Tumors

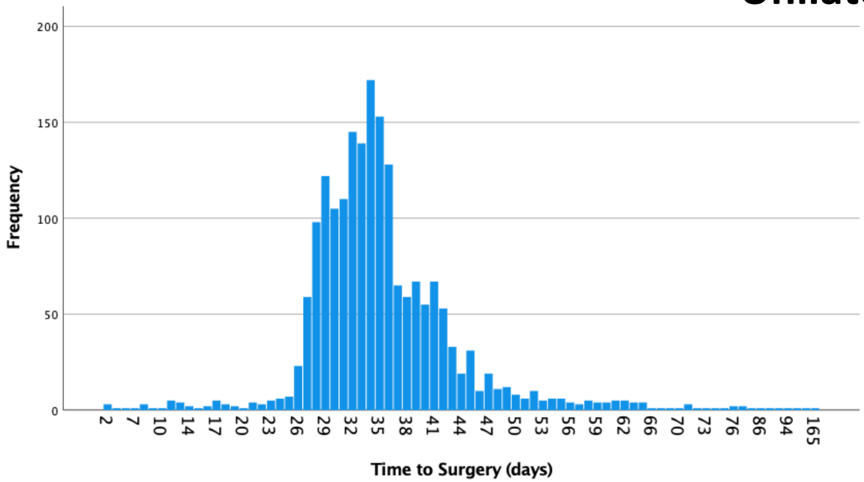

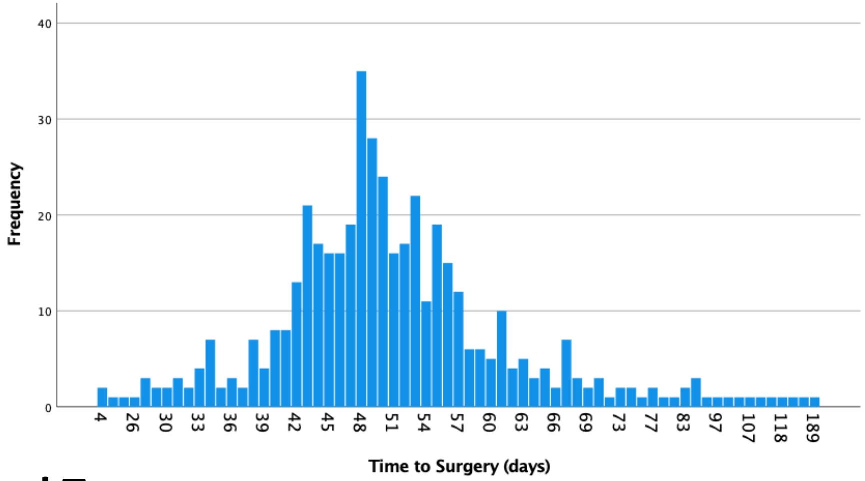

Bilateral Tumors

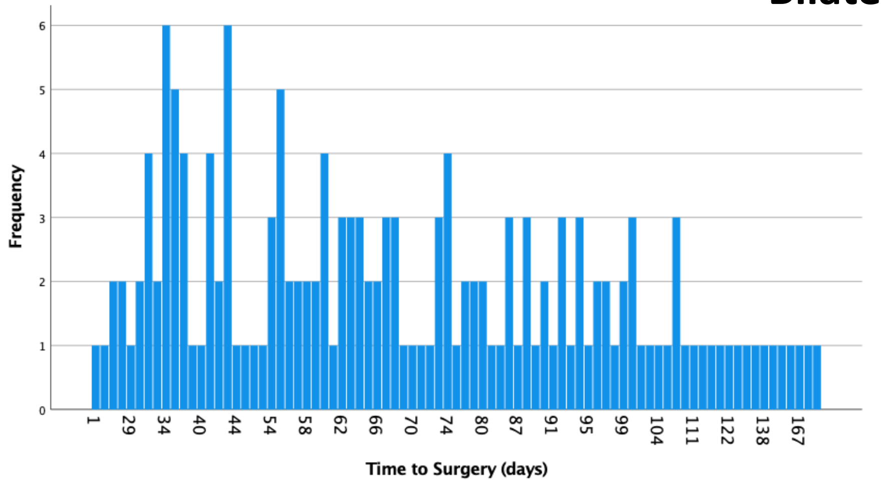

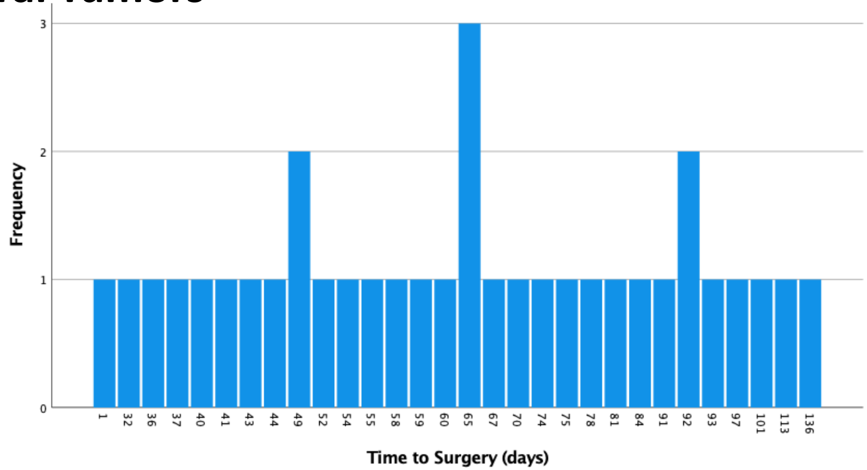

Supplement: Supplementary file 1 [file cancers-15-01494-s001.zip › Figure S1.pdf]
