# Supplementary material for: Impact of Time to Surgery on Outcome in Wilms Tumor Treated with Preoperative Chemotherapy
Source: Cancers (Basel). 2023 Feb 27;15(5):1494. doi: 10.3390/cancers15051494 (PMC10001069; doi:10.3390/cancers15051494)
Supplement: Supplementary file 1 [file cancers-15-01494-s001.zip › Figure S2.pdf]

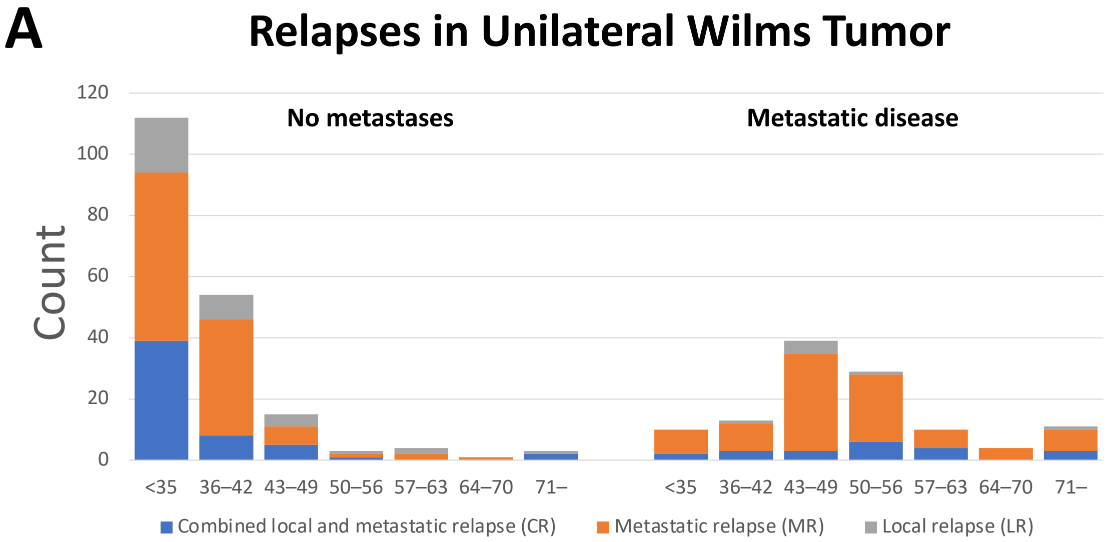

LR	18	8	4	1	2	0	1
MR	55	38	6	1	2	1	0
CR	39	8	5	1	0	0	2

0	1	4	1	0	0	1
8	9	32	22	6	4	7
2	3	3	6	4	0	3

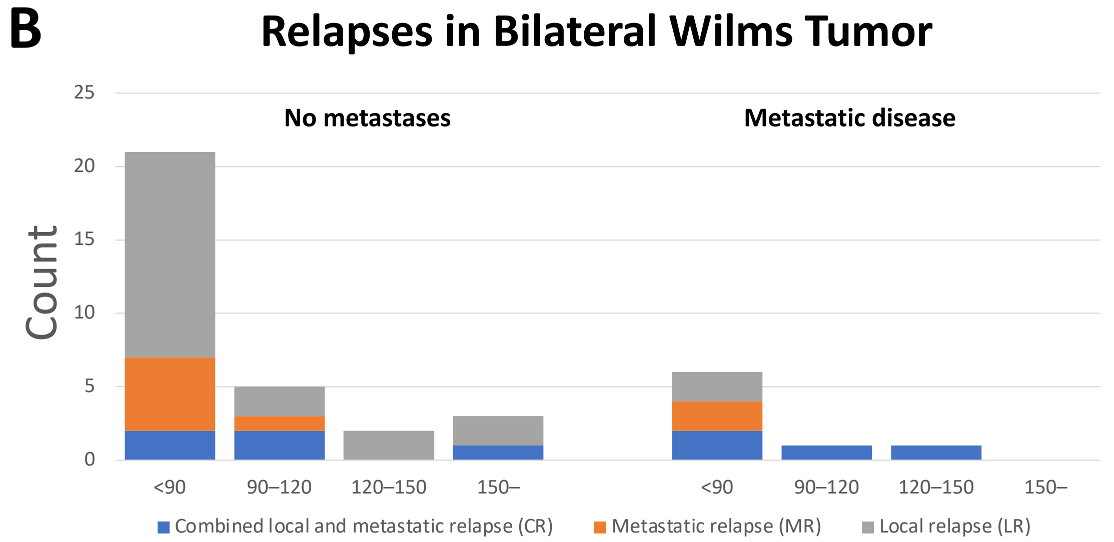

LR	14	2	2	2
MR	5	1	0	0
CR	2	2	0	1

2	0	0	0
2	0	0	0
2	1	1	0

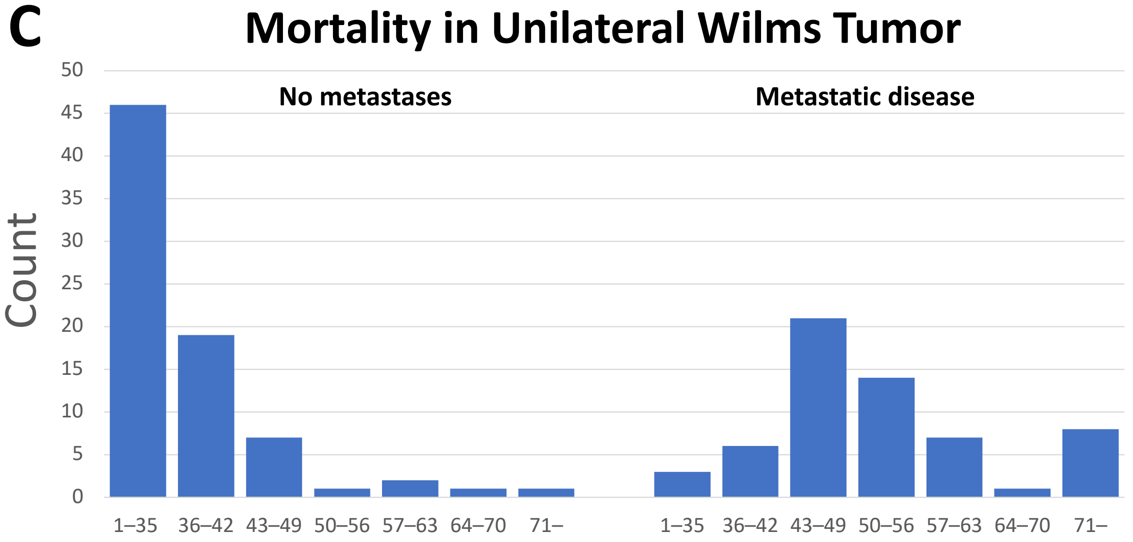

M	46	19	7	1	2	1	1

3	6	21	14	7	1	8

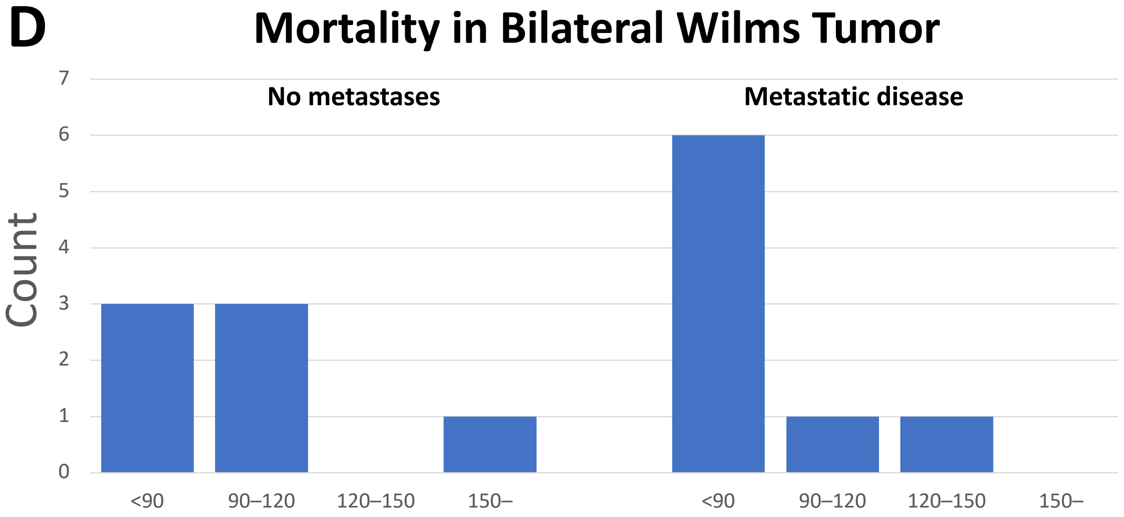

M	3	3	0	1

6	1	1	0
