# Supplementary material for: Impact of Time to Surgery on Outcome in Wilms Tumor Treated with Preoperative Chemotherapy
Source: Cancers (Basel). 2023 Feb 27;15(5):1494. doi: 10.3390/cancers15051494 (PMC10001069; doi:10.3390/cancers15051494)

Martingale Residual Plots of Overall Survival

Unilateral Wilms Tumor

A Without Metastases at Diagnosis

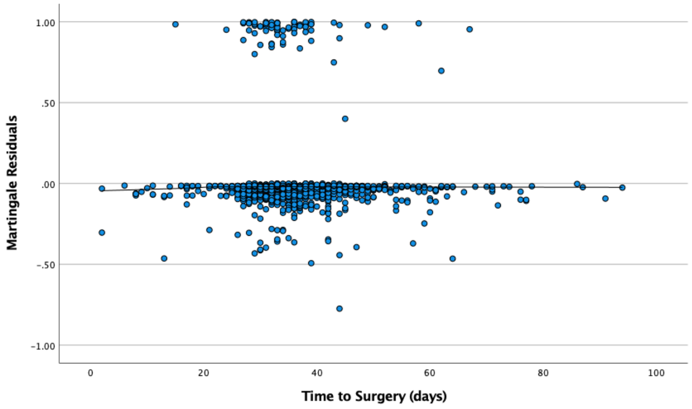

B With Metastases at Diagnosis

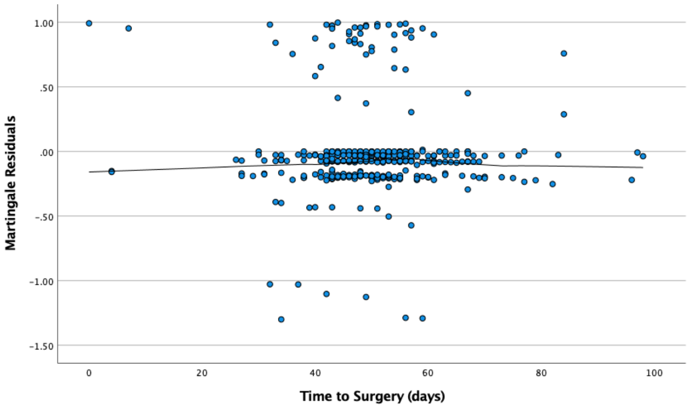

Bilateral Wilms Tumor

C Without Metastases at Diagnosis

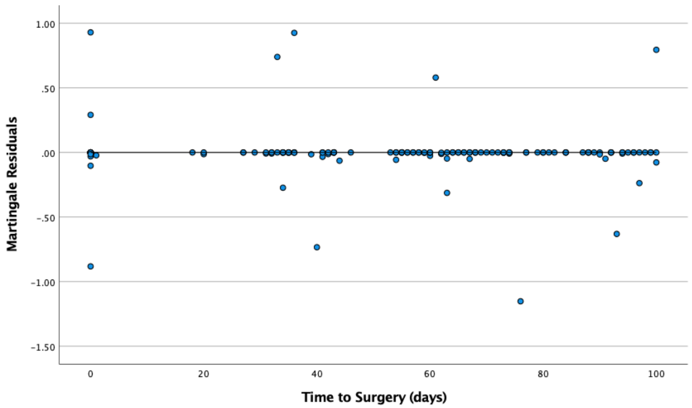

D With Metastases at Diagnosis

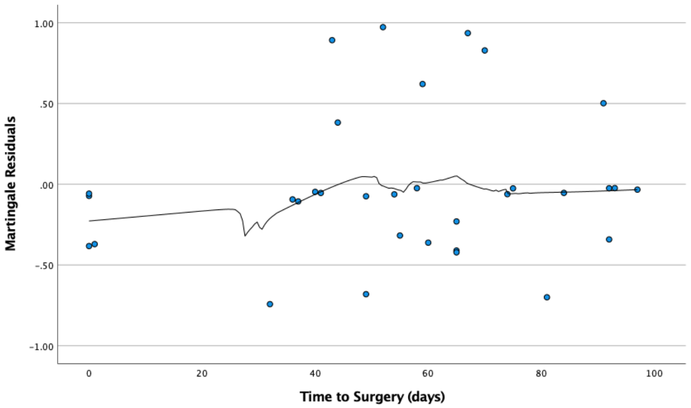

Supplement: Supplementary file 1 [file cancers-15-01494-s001.zip › Figure S6.pdf]
