# Supplementary figures and images for: Impact of Time to Surgery on Outcome in Wilms Tumor Treated with Preoperative Chemotherapy
Source: Cancers (Basel). 2023 Feb 27;15(5):1494. doi: 10.3390/cancers15051494 (PMC10001069; doi:10.3390/cancers15051494)

# Relapse-free Survival of Wilms Tumor

## Unilaterale WT

**A**

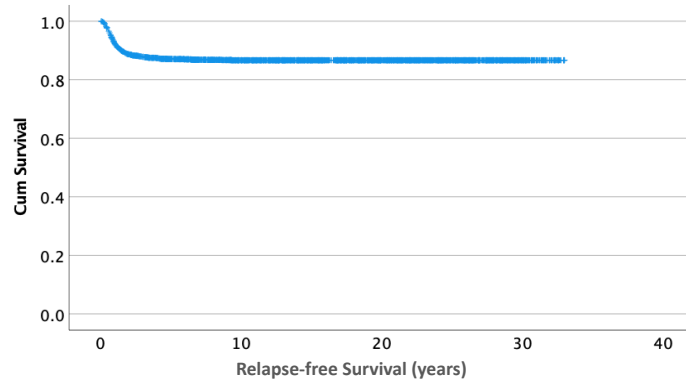

|   |      |      |      |      |
|---|------|------|------|------|
| n | 2367 | 2059 | 2059 | 2059 |
|---|------|------|------|------|

## Bilaterale WT

**B**

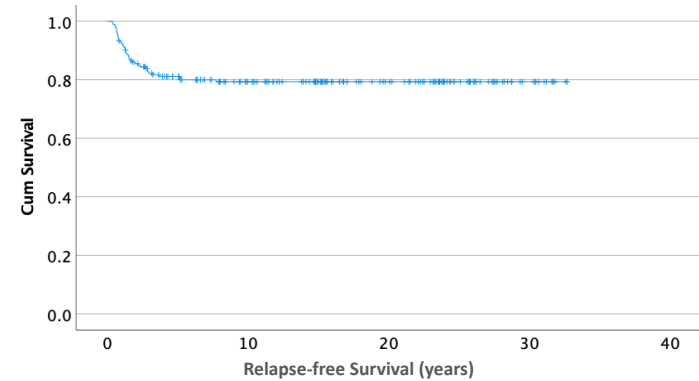

|   |     |     |     |     |
|---|-----|-----|-----|-----|
| n | 194 | 155 | 155 | 155 |
|---|-----|-----|-----|-----|

**C**

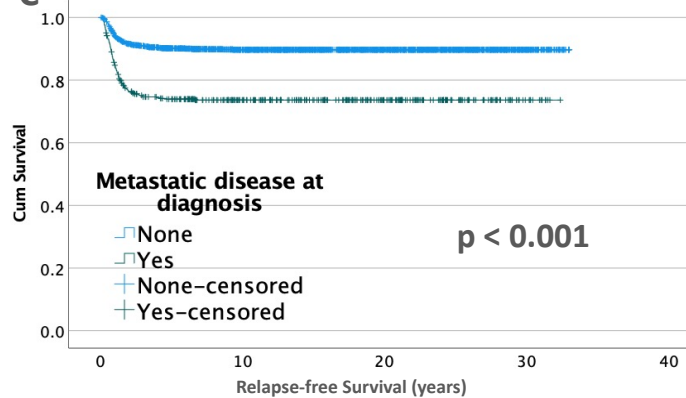

|     |      |      |      |      |
|-----|------|------|------|------|
| m - | 1918 | 1726 | 1726 | 1726 |
| m + | 449  | 333  | 333  | 333  |

**D**

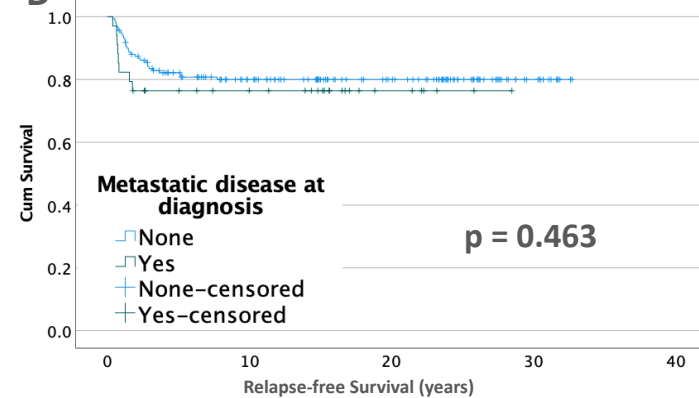

|     |     |     |     |     |
|-----|-----|-----|-----|-----|
| m - | 160 | 129 | 129 | 129 |
| m + | 34  | 26  | 26  | 26  |

Supplement: Supplementary file 1 [file cancers-15-01494-s001.zip › Figure S3.pdf]

# Overall Survival of Wilms Tumor

Unilaterale WT

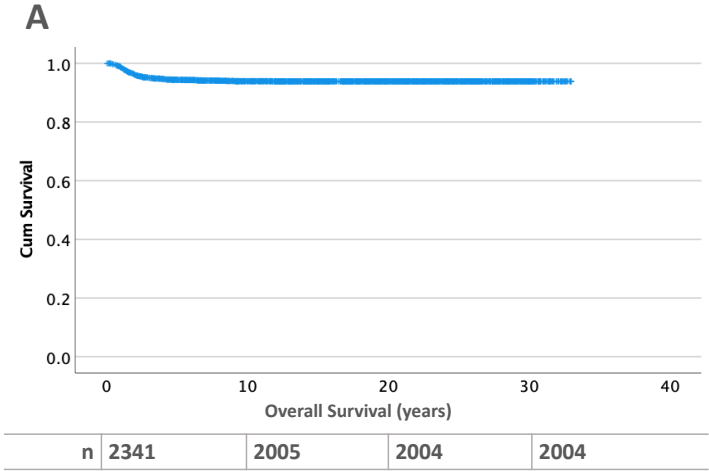

Bilaterale WT

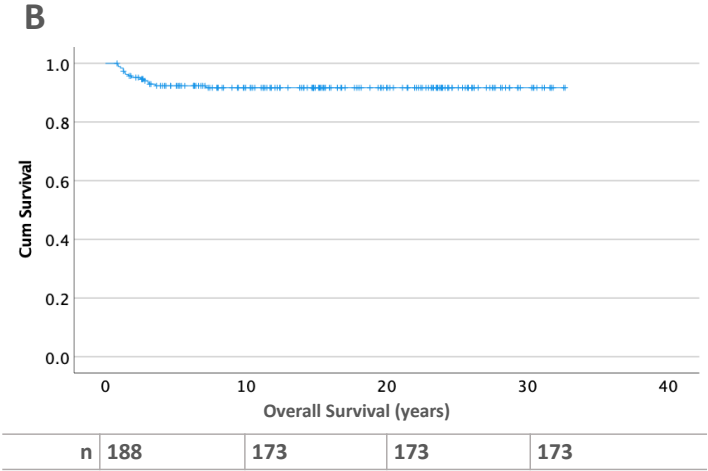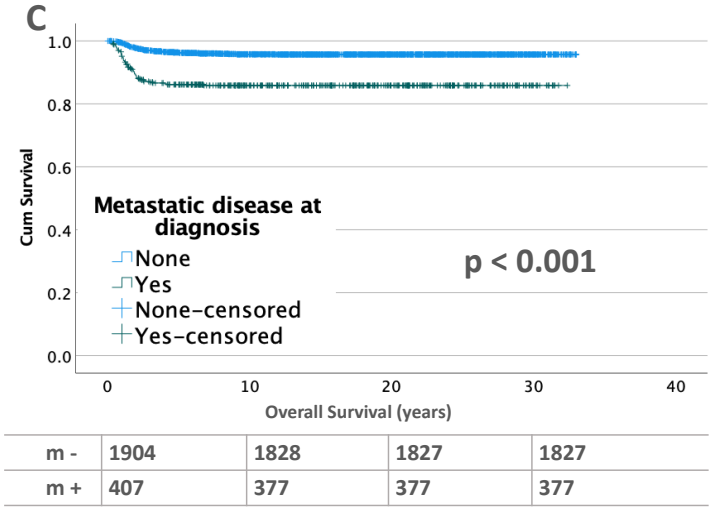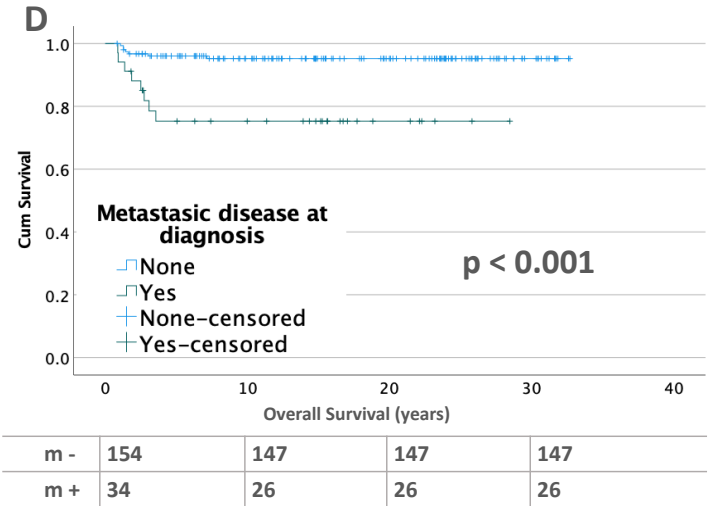

Supplement: Supplementary file 1 [file cancers-15-01494-s001.zip › Figure S4.pdf]
